# Characterization of functional brain activity and connectivity using EEG and fMRI in patients with sickle cell disease

**DOI:** 10.1016/j.nicl.2016.12.024

**Published:** 2016-12-26

**Authors:** Michelle Case, Huishi Zhang, John Mundahl, Yvonne Datta, Stephen Nelson, Kalpna Gupta, Bin He

**Affiliations:** a Department of Biomedical Engineering, University of Minnesota, USA; b Department of Medicine, University of Minnesota, USA; c Children's Hospitals and Clinics of Minnesota, MN, USA; d Institute for Engineering in Medicine, University of Minnesota, USA

**Keywords:** DMN, default mode network, SCD, sickle cell disease, EEG, electroencephalography, fMRI, functional magnetic resonance imaging, RSN, resting state networks, ICA, independent component analysis, BOLD, blood-oxygen-level dependent, PCA, principal component analysis, OBS, optimal basis set, CBA, cardioballistic artifact, MNI, montreal neurological institute, FWHM, full width at half maximum, HRF, hemodynamic response function, FDR, false discovery rate, ROI, region of interest, PCC, posterior cingulate cortex, HRF, hemodynamic response function, GLM, general linear model, SMA, supplementary motor area, ECN, executive control network, PAG, periaqueductal gray, PFC, prefrontal cortex, Sickle cell disease, Pain, Resting state networks, Functional MRI, EEG

## Abstract

Sickle cell disease (SCD) is a red blood cell disorder that causes many complications including life-long pain. Treatment of pain remains challenging due to a poor understanding of the mechanisms and limitations to characterize and quantify pain. In the present study, we examined simultaneously recording functional MRI (fMRI) and electroencephalogram (EEG) to better understand neural connectivity as a consequence of chronic pain in SCD patients. We performed independent component analysis and seed-based connectivity on fMRI data. Spontaneous power and microstate analysis was performed on EEG-fMRI data. ICA analysis showed that patients lacked activity in the default mode network (DMN) and executive control network compared to controls. EEG-fMRI data revealed that the insula cortex's role in salience increases with age in patients. EEG microstate analysis showed patients had increased activity in pain processing regions. The cerebellum in patients showed a stronger connection to the periaqueductal gray matter (involved in pain inhibition), and negative connections to pain processing areas. These results suggest that patients have reduced activity of DMN and increased activity in pain processing regions during rest. The present findings suggest resting state connectivity differences between patients and controls can be used as novel biomarkers of SCD pain.

## Highlights

• Simultaneous EEG-fMRI recordings revealed altered connectivity in sickle cell patients.
• Reduced activity observed in default mode network and executive control network.
• Patients' salience network strength increases with age; opposite seen in controls.
• EEG-fMRI parameters reflect disease severity in sickle cell patients.

## 1 Introduction

Sickle cell disease (SCD) is an inherited blood disorder that can result in life-long pain (Platt et al., 1991). This disorder causes red blood cells to deform into sickle shapes with poor oxygen carrying ability leading to recurrent ischemia-reperfusion injury, end-organ damage, and pain (Rees et al., 2010). In SCD, treatment of pain is challenging because pain episodes can start in infancy and progressively increase throughout life, causing chronic pain. Moreover, recurrent episodes of acute pain requires hospitalization and impairs quality of life (Platt et al., 1991). Opioids remain the mainstay of analgesic therapy for chronic and acute pain (Ballas et al., 2012). However, patients are often recalcitrant to opioid therapy, can be denied treatment due to “opioidphobia”, or are over-treated. Recently, some critical peripheral and spinal mechanisms underlying pain have been recognized using humanized sickle mouse models (Cataldo et al., 2015, Hillery et al., 2011, Kohli et al., 2010, Valverde et al., 2016, Vincent et al., 2015).

Recent observations suggest that “central sensitization” may underlie chronic pain due to constitutive sensitization of spinal dorsal horn neurons in sickle mice (Cataldo et al., 2015). However, the understanding of neural pathways and activities in the brain influenced by pain remain an unmet need in SCD. It is a challenge to examine the mechanisms in SCD patients due to disease heterogeneity and unpredictable episodes of acute pain. We hypothesized that non-invasive imaging methods, such as electroencephalography (EEG) and functional magnetic resonance imaging (fMRI) (He et al., 2011, He and Liu, 2008, Michel and He, 2011), can be utilized in order to better understand the mechanisms of pain in SCD and develop methods to quantify and characterize pain in patients. Non-invasive imaging has been utilized in patients with epilepsy to localize seizure onset zones by recording resting state data in either fMRI or EEG (Lu et al., 2014, Zhang et al., 2015), and hence these same tools can be applied to chronic pain to find alterations in brain activity. A recent study showed altered neural connectivity in the brain of SCD patients using fMRI (Darbari et al., 2015).

Functional brain imaging studies have suggested that during resting state the brain is active and forms patterns of activity called resting state networks (RSN) (Fox et al., 2005). Certain RSN have been identified using fMRI, including the default mode network (DMN), salience, sensory motor, and attention (Farmer et al., 2012, Raichle et al., 2001). Altered DMN activity has been observed in a number of neurological disorders and chronic pain (Buckner et al., 2008, Kucyi et al., 2014, Loggia et al., 2013). These abnormalities in functional connectivity suggest that chronic pain conditions alter resting state activity.

In our study, we used traditional fMRI methods to assess altered connectivity in RSN. These methods included independent component analysis (ICA) and seed-based analysis (Correa et al., 2007, Greicius et al., 2009, Moeller et al., 2011, Vollmar et al., 2012). EEG dynamics can be extracted by selecting unique EEG features and comparing their time courses to fMRI data (He and Liu, 2008, Liu and He, 2008). EEG-fMRI methods have been developed to study resting state in healthy subjects using techniques in frequency, spatial, and time domains. To have a more mechanistic understanding of how RSN are linked to neurophysiological manifestations, we included concurrent EEG in our study. EEG has high temporal resolution and direct measurement of underlying neurological activity. There have been previous studies deploying EEG-fMRI to study DMN in healthy subjects (Hlinka et al., 2010, Laufs et al., 2003a, Laufs et al., 2003b, Mantini et al., 2007). Our data show that non-invasive EEG-fMRI methods are well tolerated by patients with SCD without any adverse events; and that comparison with RSN activity in healthy subjects can be used to examine abnormalities in RSN activity in patients with SCD.

## 2 Methods

### 2.1 Healthy subjects

We recruited 15 healthy volunteers, 8 of which were female. The mean age and standard deviation were 28.8 ± 10.7 years, respectively. The healthy subjects' ethnicity included 6 African Americans, 1 Hispanic, 1 Asian, and 7 Caucasian. All subjects met the MR safety criteria and gave their written informed consent. None of the subjects reported any previous neurological or psychiatric disorders, psychoactive medication or history of drug abuse. The study was approved by the Institutional Review Boards of the University of Minnesota.

### 2.2 SCD patients

A total of 15 patients with SCD were recruited to participate in the study. There were 7 female patients, and the mean age and standard deviation was 24.5 ± 6.8 years, respectively. There were 2 patients that were under the age of 18 in this study. Patients were asked to rate their pain on the day of the study on a scale of 0 to 10 (with 10 being the worst pain imaginable) and were allowed to take all of their usual medications, including narcotic pain medications. A MR screening form was completed to ensure patients could safely participate in the study. All patients participated in this study with written consent according to a protocol approved by the Institutional Review Board of the University of Minnesota. For the minor participants, written assent was obtained as well as consent from a parent or legal guardian. Patients were also asked if they were willing to share their medical records with the research staff. An additional informed consent was obtained for all patients willing to share their medical records. One patient was not comfortable sharing their medical records.

### 2.3 EEG recording

**Fig. 1:**
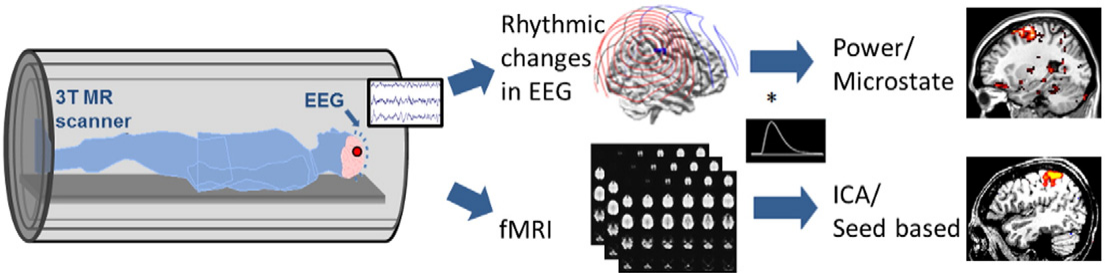
Schematic diagram of data analysis steps conducted on simultaneously recorded EEG and fMRI data. Power and microstate analysis were performed on EEG data and the resulting time courses were convolved with the hemodynamic response function (HRF) to obtain activation maps. Independent component analysis (ICA) and seed based analysis were performed on fMRI data to find activation maps.

A 64-channel MR-compatible EEG cap was placed on the subject's scalp. One electrode was placed on the subject's back to record cardiac activity for noise removal purposes later on. Electrode impedances were brought below 20 kΩ. The EEG was amplified using MR-compatible amplifiers (BrainAmp MR 64 plus, BrainProducts, Germany) and recorded at 1000 Hz. Recordings of EEG were done outside the scanner for all controls and all patients. EEG was recorded both inside and outside of the scanner on the same day of the experiment for 11 patients and 13 controls. For the remaining patients and controls, fMRI was obtained separately from EEG recordings. During outside scanner recordings, the subject was told to sit still and rest with eyes open in a private room. Outside scanner recording lasted 20 min. During inside scanner recording, subjects were asked to have their eyes open and lie still in the scanner and not to fall asleep. Fig. 1 shows the schematic diagram of the experimental procedure for EEG-fMRI. Each recording lasted for 8 min. We collected at least a total of 20 min of simultaneous EEG-fMRI for each subject that received this procedure.

### 2.4 MRI recording

We used a 3 T Siemens Magnetom Trio scanner (Erlangen, Germany) with 16 channel head coil for all but one subject. The last subject's data was obtained on a 3 T Siemens Magnetom Prisma scanner (Erlangen, Germany) with a 20 channel head coil due to upgrades being conducted at the Center for Magnetic Resonance Research at the University of Minnesota. Individual anatomical MRI data were collected which consisted of 240 contiguous sagittal slices with 1 mm slice thickness (matrix size: 256 ∗ 256; FOV: 256 mm ∗ 256 mm; TR/TE = 20 ms/3.3 ms) on a 3 T MRI system (Siemens, Erlangen, Germany). Additionally, each subject was instructed to lie quietly in the scanner for two to three functional scans, each lasting 8 min. Whole-brain functional images with blood-oxygen-level dependent (BOLD) contrast were acquired using gradient echo planar imaging sequence (40 axial 3-mm thick sequential slices with 0.3-mm gap; TR/TE = 2500 ms/30 ms; flip angle = 90°; matrix size: 64 ∗ 64; FOV: 192 mm ∗ 192 mm).

### 2.5 EEG preprocessing

The MR gradient artifact was removed using a principle component analysis (PCA)-based optimal basis set (OBS) algorithm (Niazy et al., 2005). ECG recordings which were collected from the electrode placed on the subject's back were used to detect and remove the cardioballistic artifact (CBA). The timing of each heartbeat artifact in the channel was determined using an R-peak detection algorithm adapted from (Liu et al., 2012a). The final artifact correction procedure is based on a combination of ICA, OBS, and an information-theoretic rejection criterion (Liu et al., 2012a). In this process the signal is decomposed into independent components, which are rejected if the mutual information between the component's time course and the CBA artifact is sufficiently high. The remaining components are then divided into epochs around each heartbeat and an optimal basis set is obtained across all epochs to fit and remove the artifacts. Detection of bad electrodes and data epochs was performed before CBA detection, and again after CBA correction. Electrodes were first re-referenced to a common average of electrodes connected to the same amplifier, and then to the combined average. Together with EEG data obtained from outside of MR scanner, the EEG signal was filtered and down-sampled to 256 Hz.

### 2.6 fMRI preprocessing

All fMRI data were pre-processed for slice scan time correction, 3-D motion correction and temporal filtering using SPM8 software (Ashburner et al., 2012). All brains were aligned to the anterior-posterior commissural line and normalized by transformation into MNI space (Montreal Neurological Institute). The fMRI data were then spatially co-registered to the structural MRI. Smoothing was performed in SPM8 to three times the size of the original voxels using full width at half maximum (FWHM). The first 10 images from each fMRI session were removed to ensure all fMRI data had reached a steady state of excitation.

### 2.7 BOLD signal comparison

**Fig. 2:**
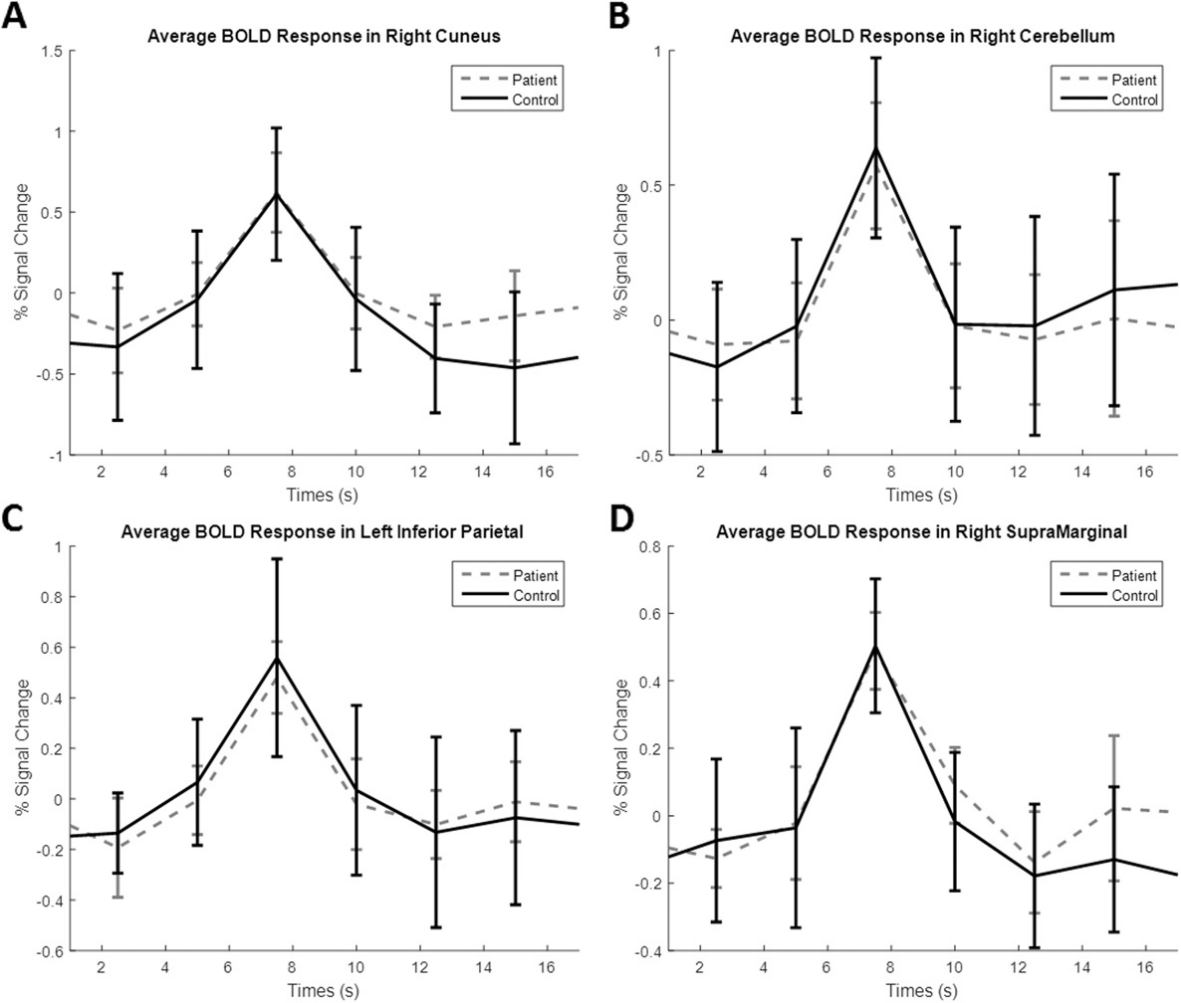
Blood-oxygen-level dependent (BOLD) responses in terms of percent signal change across time for both patients and controls. A. Average BOLD responses located in the right cuneus region. B. Average BOLD responses located in the right cerebellum. C. Average BOLD responses located in the left inferior parietal cortex. D. Average BOLD responses located in the right supramarginal gyrus. Responses from four of the eight regions tested are shown here. No region showed significant differences between patients and controls.

A major concern of this study was whether the fMRI data from patients would accurately reflect neurological activity due to the nature of their disease. The BOLD signal measures changes in blood oxygenation. SCD directly affects red blood cells and this could induce changes in BOLD signals that do not reflect neural activity. In order to determine if alterations observed in fMRI reflect true neural activity or abnormalities in blood flow caused by SCD we compared the hemodynamic response function (HRF) of patients and controls. The spontaneous HRF was obtained from resting state data using a previously described method (Wu et al., 2015, Wu et al., 2013). The raw BOLD signal was obtained from several brain regions using MarsBaR (Brett et al., 2002). The percent signal change was calculated for each BOLD time course across all subjects. The spontaneous HRF was found by setting a peak threshold to at least 0.6% signal change. The average HRF were found for both controls and patients and are plotted in Fig. 2. The peak widths and peak prominence for each region were recorded and displayed in Table 1. No significant differences between controls and patients were found in any of the regions studied. Since the HRF seems stable in patients, we believe this suggests that fMRI data reflects neural activity rather than abnormalities in vasculature in patients. It should also be noted that many patients were in no to little pain during our experiments, meaning they were in a relatively stable condition. Recent studies have also shown that during resting state, SCD patients have increased cerebral blood flow compared to healthy controls to compensate for decreased oxygen levels so that oxygen delivery at rest is normal (Bush et al., 2016, Jordan et al., 2016). This suggests fMRI measurements should be normal during rest in SCD patients.

**Table 1:** Characteristics of hemodynamic response function (HRF) for patients and controls in different brain regions.

HRF characteristic	Peak width (s)			Peak prominence (% change)		
ROI	Patients	Controls	p-Value	Patients	Controls	p-Value
Cuneus	5.111293	5.655936	0.255852	1.157787	1.436841	0.137429
Fusiform	5.899524	5.229846	0.195902	0.996871	1.246177	0.070719
Inferior parietal	5.633893	5.763587	0.713935	0.906336	1.086704	0.253439
Midcingulate	5.746368	5.947603	0.592804	0.988434	1.236614	0.105184
Midoccipital	5.825247	5.762134	0.891424	0.865477	1.271622	0.061947
Cerebellum	5.295872	5.170938	0.770602	1.112825	1.165956	0.735398
Insula	5.188698	5.566975	0.318844	0.949436	1.112952	0.115258
Supramarginal gyrus	5.634376	5.578053	0.903875	0.960899	1.121443	0.175291

Summary of BOLD analysis results showing the average peak width and average peak prominence of the hemodynamic response function (HRF) of patients and controls. Eight randomly selected regions in the brain were chosen to detect if there was any significant differences in the HRF characteristics. The peak width is measured in seconds, and the peak prominence is measured in percent change.

### 2.8 Independent component analysis of fMRI data

ICA in the spatial domain was performed using the Group ICA of fMRI toolbox (GIFT) (Rachakonda et al., 2007). Detailed methodological principles of ICA decomposition using blind source separation, the method implemented in GIFT, were previously described (Correa et al., 2007, Correa et al., 2005). First, PCA was applied to data from each subject to reduce the computational cost of the analysis. Thirty independent components were computed using the infomax algorithm. Back reconstruction computed spatial maps and time courses for each component for all subjects. The voxel intensities of each independent component mean group maps were converted to z-scores. A higher z-score represents a higher correlation coefficient. The ratio of low frequency power to high frequency power was calculated for each component. This ratio was used to help determine if the component was noise. The higher the ratio value, the more likely the component is an acceptable signal. A threshold of p = 0.05 corrected for false discovery rate (FDR) was applied to group average maps.

The “spectral group compare” toolbox in GIFT was used to calculate the power density to evaluate the frequency distribution of the time courses for each RSN. The frequency range was spaced into three equally spaced bins between 0 and 0.25 Hz at 0.8 Hz intervals. Control and patient comparison was done using one-sample *t*-tests for each frequency bin.

### 2.9 Seed based analysis

Connectivity was assessed using the CONN functional connectivity toolbox (Whitfield-Gabrieli and Nieto-Castanon, 2012). Functional and anatomical data for each subject was loaded into the toolbox. First-level analysis was done using all regions of interest (ROIs) listed in the CONN toolbox to perform individual ROI to ROI and ROI to voxel analysis for all subjects. Second-level analysis was done for control and patient groups. Connectivity ROI to ROI maps were plotted showing connection strength and polarity, and a p-value of 0.05 (FDR corrected) was used to threshold the maps. ROI to ROI maps were used to identify important seeds that showed altered connectivity between control and patient groups. The right cerebellum Crus I seed was identified as having significant negative connectivity differences between patients and controls and this seed was used in a ROI to voxel analysis. The ROI to voxel analysis used a p-value of 0.05 (FDR corrected) to threshold the significant clusters with negative connectivity to the seed region in patients. The connectivity to these regions was obtained in both controls and patients to validate significant differences between the two groups. The connectivity values of the patients were also compared to the clinical data to find any correlations between connectivity and disease severity.

### 2.10 EEG-informed fMRI

**Fig. 3:**
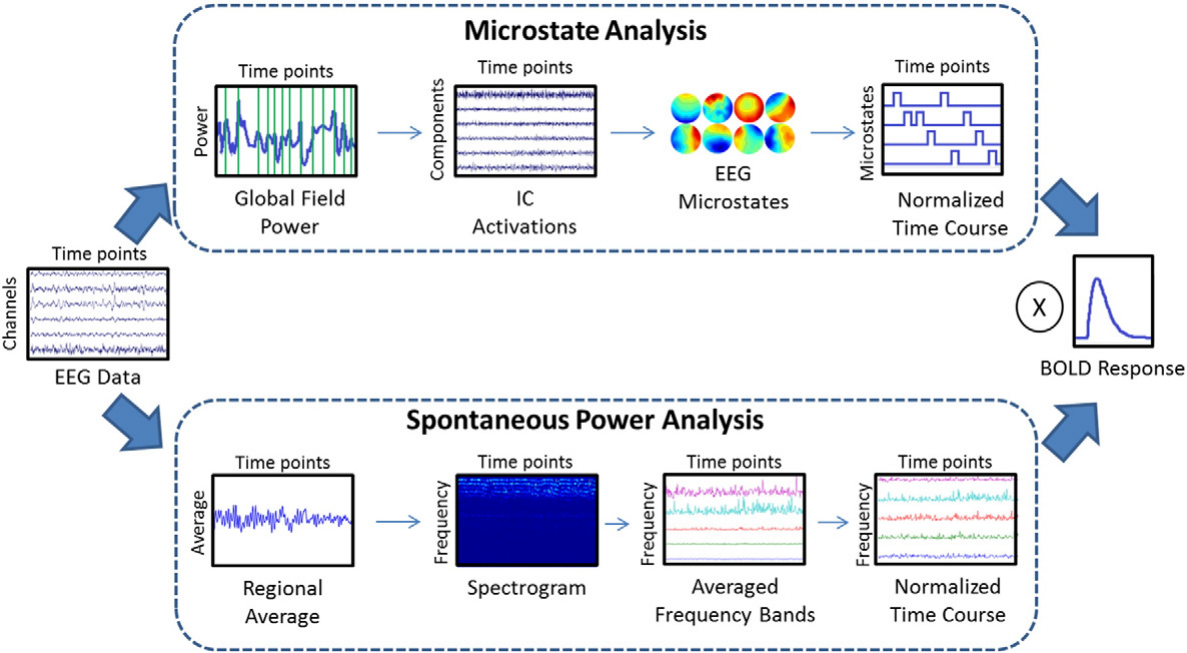
Graphic diagram of data analysis steps for simultaneous EEG-fMRI methods. The preprocessed EEG data were analyzed using two methods. The top chart shows the microstate analysis method. The peak points of the global field power were concatenated together to identify thirty independent components using ICA. After eliminating noisy components, nine group microstates were identified for further analysis. The time course for each microstate was normalized so that only one microstate would be active for any given time point. The bottom chart shows the basic steps for spontaneous power analysis. An average time course was determined from a specific brain region. The averaged time course was used to construct a spectrogram so that the average time course for EEG frequency bands could be found. The frequency band time courses were mean-scaled so that their ranges would be comparable to each other. Finally, the normalized time courses were convolved with the hemodynamic response function to be used as regressors in a general linear model with fMRI data.

Spontaneous power fluctuations over different frequency bands were calculated from EEG data. Frequency bands include delta (2–4 Hz), theta (5–7 Hz), alpha (8–12 Hz), beta1 (13–21 Hz), and beta2 (22–30 Hz). The method for obtaining power fluctuations was previously discussed (Laufs et al., 2003a, Laufs et al., 2003b). An averaged time course was obtained from the occipital electrodes, which best reflects alpha activity. Enhancing alpha activity was done because the DMN has been linked to this frequency band (Knyazev et al., 2011, Laufs et al., 2003a, Laufs et al., 2003b, Mantini et al., 2007). The average time course was used to build a spectrogram. The calculated power values were averaged across the specified frequency bands within the spectrogram and mean-scaled to standardize the range of each frequency band to get the final power fluctuation time courses; this method is shown in the bottom path of Fig. 3. An EEG microstate analysis was also performed using methods previously described (Yuan et al., 2012). A total of 30 microstates were calculated for the entire group by finding the peaks in the global field power, and the noisy ones were eliminated from further analysis. The analysis was performed by concatenating the group data together to get the final microstates. The normalized microstate time course, where only one microstate is dominant at any specific time point, was used for EEG-fMRI analysis. The top path of Fig. 3 displays the major steps of the microstate analysis method.

**Fig. 4:**
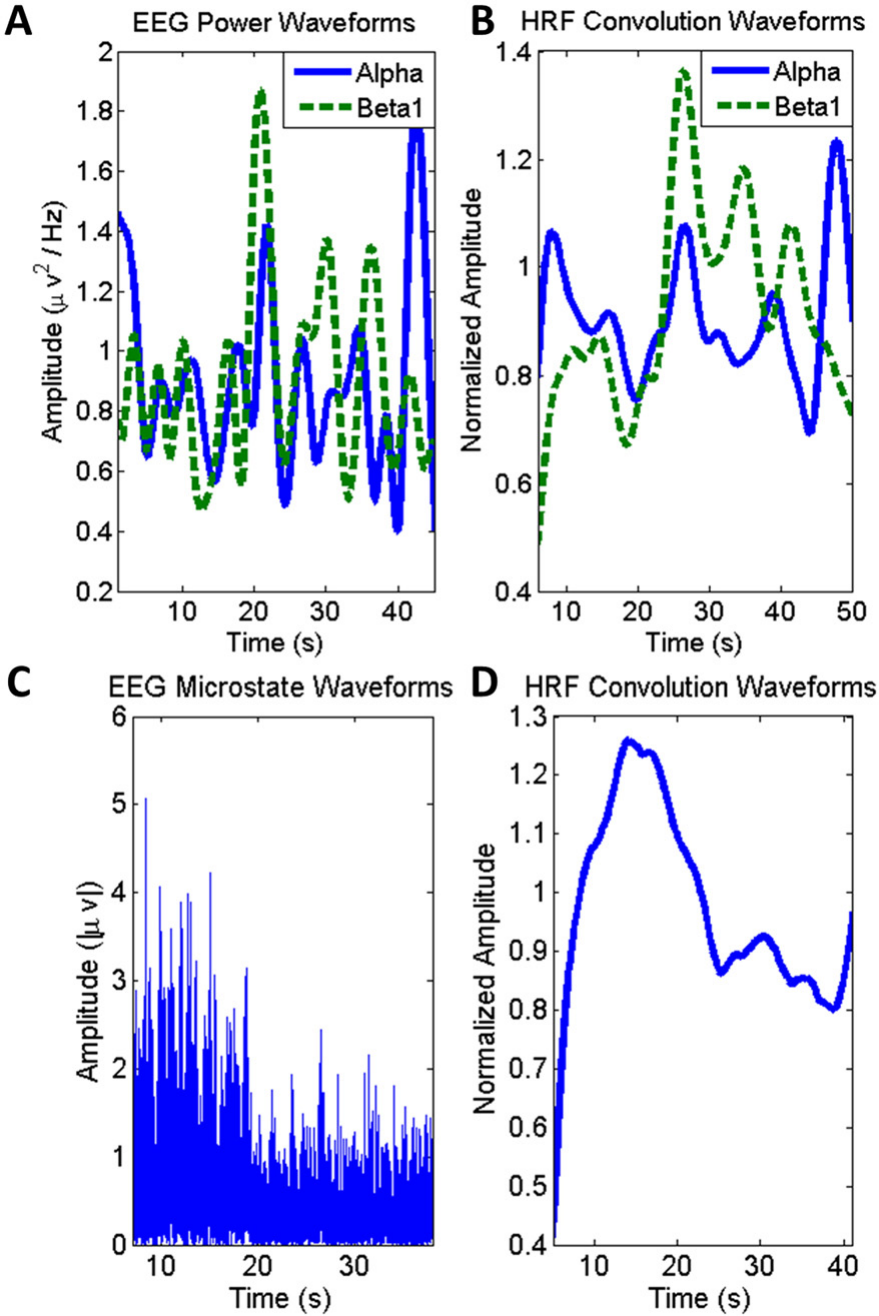
EEG time course transitions to regressors. A. Mean-scaled EEG power waveform time courses for two selected frequency bands, alpha and beta1. B. Effect of hemodynamic response function (HRF) convolution with EEG power time courses. C. Absolute intensity time course for one selected microstate. D. Effect of HRF convolution with normalized microstate time course. HRF convolved time courses (B, D) are used as the final regressors for EEG informed fMRI analysis.

EEG time courses were convolved with the HRF in order to correlate the EEG data to the fMRI results; the time courses for both methods are shown before and after HRF convolution in Fig. 4. Correlations were determined by using a general linear model (GLM). The GLM was implemented using SPM8 software. The design matrix was formed by using the convolved EEG time courses from the methods described above as well as the six rigid-body motion correction parameters as regressors. The model was estimated using the classical method in SPM8. First level analysis was used to generate single subject activation maps. For the power analysis, both a positive and negative contrast was applied to each frequency band regressor. Only a positive contrast was applied to each microstate regressor. Group analysis was performed using a second level factorial design analysis in SPM. Contrast images were obtained to compare differences between controls and patients. The total number of healthy controls was 13 and the total number of patients was 11 for group analysis. A FDR correction was applied to the group images. The p-value was set to 0.05 and these are the only voxels reported as statistically significant. Statistical maps were imported into MRIcron software for display (Rorden and Brett, 2000).

A ROI analysis was performed on the EEG-fMRI beta1 power results, where a significant cluster was observed in the “patients > controls” contrast image. The z-score of the ROI was obtained from back-projected RSN maps from our previous GIFT analysis. The salience network was chosen for the RSN due to the nature of the group activation for the beta1 band. The z-scores were obtained from both controls and patients, and compared to our clinical variables to find correlations that reflect disease severity. The average activation time of each microstate was calculated for both controls and patients by finding the total times a microstate was dominant and dividing it by the total time points within the EEG recording. A two-sample *t*-test was used to compare average activation time of each microstate between controls and patients. The average activation time was also used to find correlations with the clinical variables.

## 3 Results

### 3.1 Patient summary

We had 15 patients participate in our study with ages ranging from 16 to 38 years. The relevant clinical variables are listed in Table 2. One patient did not wish to disclose their medical records. Most patients had no pain on the day of the experiment and so the average pain score is fairly low. Due to the lack of variability, we did not find any correlations with our neural data to this parameter. Clinical parameters reflecting pain history and disease severity were used for all clinical correlation analyses. The medications used by the patients recruited for the study are listed in Table 3. The most common medications used included folate, hydroxyurea, ibuprofen, and oxycodone.

**Table 2:** Patient summary of demographics and characteristics of clinical variables.

Clinical variables	Value	
Age (years)	24.5 (± 6.8)	(N = 15)
Female (%)	47	(N = 15)
Pain score	0.73 (± 1.6)	(N = 15)
Hydroxyurea therapy (%)	57	(N = 14)
Systolic blood pressure (mm Hg)	122 (± 14)	(N = 14)
Diastolic blood pressure (mm Hg)	70 (± 10)	(N = 14)
Hemoglobin (g/dL)	10 (± 2)	(N = 14)
Hemoglobin F (%)	6 (± 6)	(N = 14)
Reticulocyte count (k/uL)	283 (± 174)	(N = 14)
White blood cell count (k/uL)	10 (± 3)	(N = 14)
Platelet count (k/uL)	295 (± 90)	(N = 14)
Chronic red cell transfusion (%)	14	(N = 14)
Emergency room visits in past 2 years	11 (± 14)	(N = 14)
Hospitalizations in past 2 years	7 (± 7)	(N = 14)

Sickle cell type	%	N
Hemoglobin SS	57	8
Hemoglobin SC	21	3
Hemoglobin SB + thalassemia	7	1
Hemoglobin SB0 thalassemia	14	2

Summary of recruited patients' demographic and clinical variable information. Values are mean ± standard deviation unless specified otherwise. N indicates the number of patients included for the summary.

**Table 3:** Summary of all medications used by recruited patients.

	%	N
Medications		
Albuterol inhaler	21	3
Aspirin	7	1
Benztropine	7	1
Celebrex	7	1
Clozapine	7	1
Deferasirox	14	2
Desyrel	7	1
Diphenhydramine	14	2
Escitalopram	7	1
Folate	57	8
Fondaparinux	7	1
Gabapentin	21	3
Haldol	7	1
Hydroxyurea	57	8
Ibuprofen	57	8
Meloxicam	14	2
Topiramate	7	1
Zolpidem	7	1

Narcotic pain medications		
Dilaudid	7	1
Methadone	7	1
Morphine	7	1
MScontin	14	2
Oxycodone	57	8
Tramadol	14	2

Summary of recruited patients' medication. N indicates the number of patients using the type of medication. Several patients were taking more than one type of medication.

### 3.2 Resting state networks using ICA

**Fig. 5:**
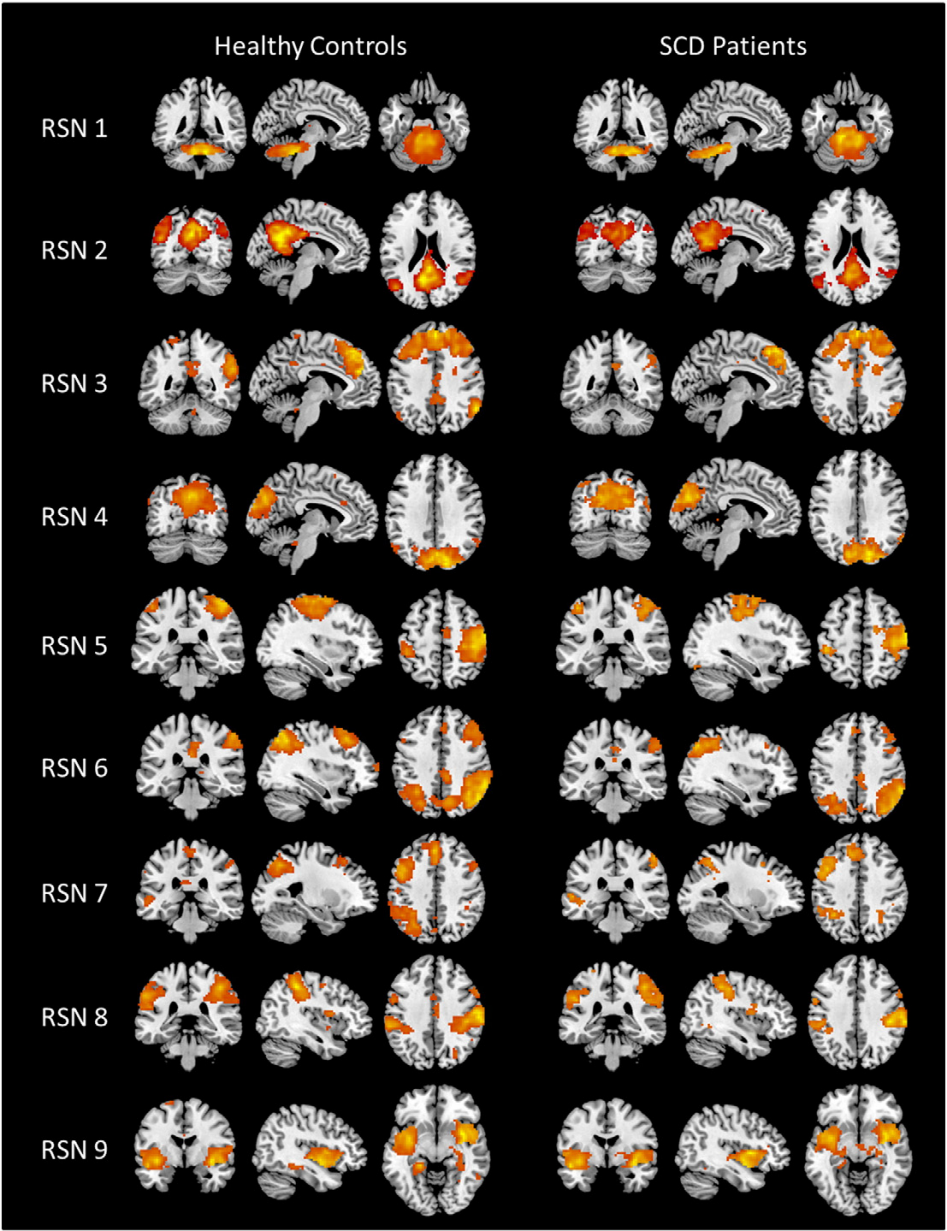
Resting state networks (RSN) identified using ICA for healthy controls and SCD patients. RSN1 is the cerebellum network. RSN2 is the posterior DMN. RSN3 is the anterior DMN. RSN4 is the visual processing network. RSN5 is the sensory motor network. RSN6 is the right executive control network. RSN7 is the left executive control network. RSN8 is the dorsal attention network. RSN9 is the salience network.

Fig. 5 displays nine identified RSN detected by the ICA approach in controls and patients. The RSN found in our subjects are similar to RSN identified in past studies (Di and Biswal, 2014, Heine et al., 2012, Lee et al., 2013, Van den Heuvel and Hulshoff Pol, 2010). RSN1 corresponds to the cerebellum network with activity mainly in the cerebellum. RSN2 and RSN3 relate to the posterior and anterior DMN, respectively. The DMN activity is found in precuneus/posterior cingulate cortex (PCC), medial frontal cortex, and inferior parietal regions, with either posterior or anterior localization. The visual network with activation in the occipital cortex is RSN4. RSN5 has activity in the primary motor cortex (precentral gyrus), primary somatosensory cortex (postcentral gyrus), and supplementary motor area (SMA), which corresponds to the sensory motor network. The right and left executive control network (ECN) are shown in RSN6 and RSN7, respectively. The superior parietal and superior frontal regions, either right or left lateralized, were active for the ECN. RSN8 was identified as the dorsal attention network with activity mainly in the intraparietal sulcus. Finally, RSN9 is related to the salience network with activity in bilateral insular cortex.

**Fig. 6:**
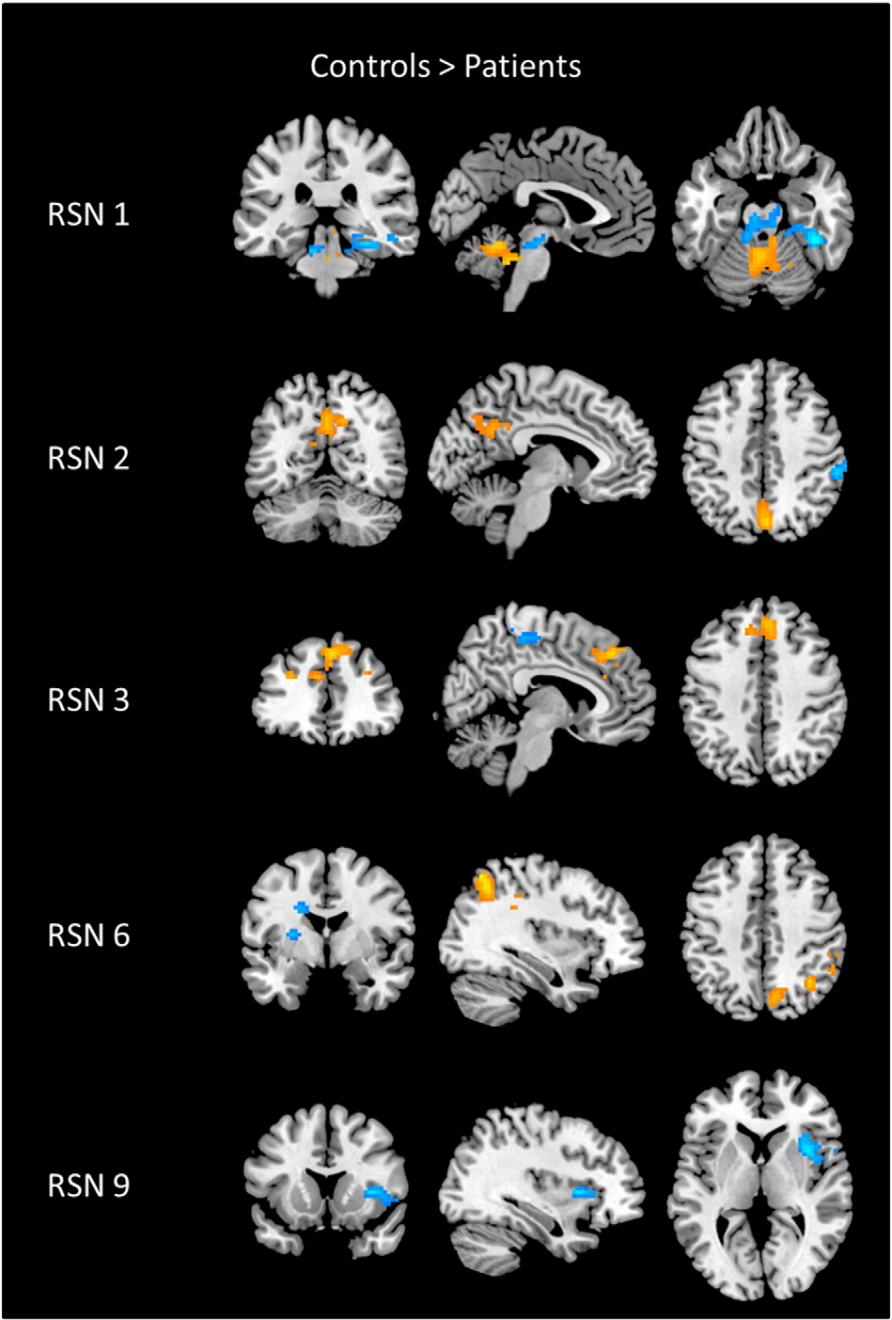
Contrast images of resting state networks (RSN) showing patients subtracted from the healthy controls. The blue color shows activation areas where patients have more connectivity, and the yellow color shows activation areas where controls have more connectivity. Contrast image of RSN1 shows significant differences observed in the cerebellum for controls, and in the periaqueductal gray matter for patients. Contrast image of RSN2shows significant differences observed in posterior cingulate cortex for controls, and in supramarginal gyrus for patients. Contrast image of RSN3 shows significant differences observed in medial frontal cortex for controls, and in the paracentral lobule for patients. Contrast image of RSN6 shows significant differences observed in posterior parietal cortex for controls, and in basal ganglia for patients. Contrast image of RSN9 shows significant differences observed in right insula cortex for patients.

The contrast images of RSN1, RSN2, RSN3, RSN6 and RSN9 are shown in Fig. 6. The group result of patients' RSN is subtracted from the group result of controls' RSN, where orange shows regions where controls have greater connectivity and blue shows regions where patients have greater connectivity. For RSN1, the controls have greater connectivity to the cerebellum, but patients have more connectivity to the periaqueductal gray (PAG), a major node in the descending pain pathway (Lee and Tracey, 2013, Vanegas and Schaible, 2004). The contrast images show that controls have stronger connectivity to DMN regions for both RSN2 and RSN3, with greater connectivity in the PCC and the medial frontal cortex, respectively. Patients had greater connectivity in the supramarginal gyrus for the posterior DMN and greater connectivity in the paracentral lobule for the anterior DMN. Controls had greater connectivity in precuneus, and right posterior parietal cortex for RSN6, where patients had greater connectivity to the basal ganglia. For RSN9, only the patients showed greater connectivity in the right insula cortex.

**Fig. 7:**
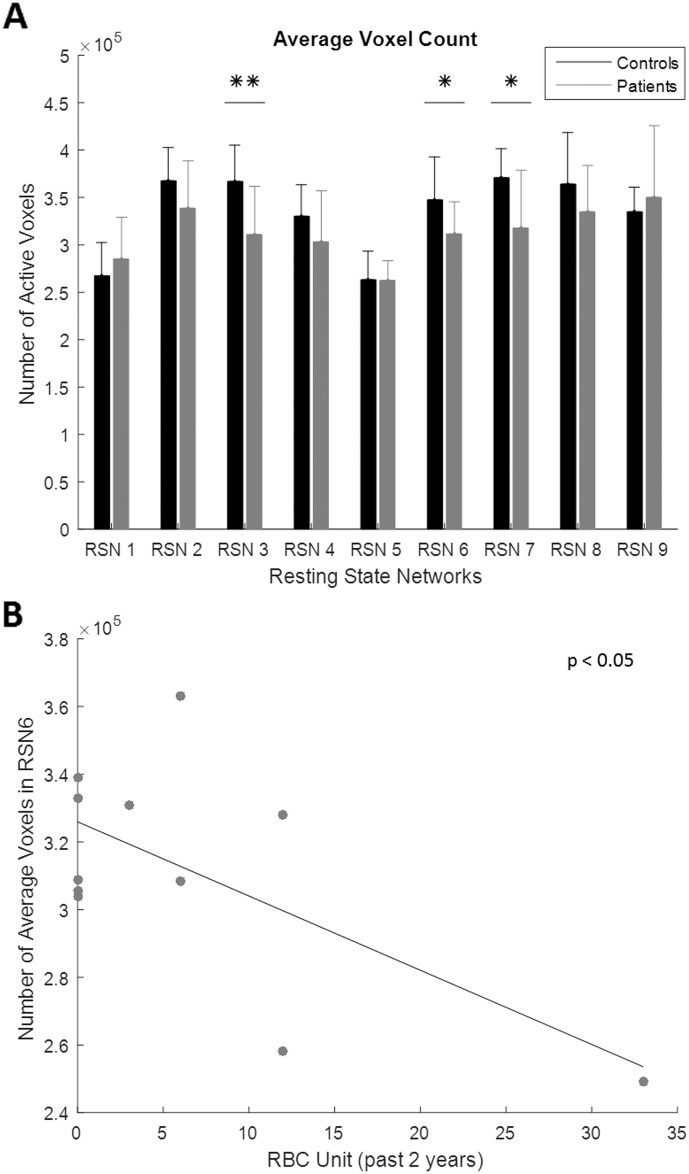
Statistical analysis of voxel count for identified resting state networks (RSN). A. Bar plot of the average number of active voxels for healthy controls and patients across all resting state networks identified from ICA. The * shows significance where p < 0.05, and the ** shows significance where p < 0.01. Standard deviation is displayed using error bars. B. Scatter plot showing relationship of number of active voxels for RSN6 and the amount of transfused red blood cells. The linear trend line is plotted and the significance of the relationship is shown to be p < 0.05. The less amount of voxels active in RSN6 correlates to greater transfused red blood cells which indicates increased disease severity.

The average number of significant voxels was computed for all RSN activation maps for both control and patient groups, shown in Fig. 7A. Overall, controls tended to have more active voxels than patients, where patients had more voxels than controls only in RSN1 and RSN9. The only significant differences were found to be in RSN3 (p-value = 0.007), RSN6 (p-value = 0.038), and RSN7 (p-value = 0.020). A negative correlation was found between the number of active voxels in RSN6 and the amount of transfused red blood cells, shown in Fig. 7B. A greater amount of red blood cell units indicates a worse disease severity. The executive control had significantly reduced activity in SCD patients, and this deficit was linked to disease severity.

### 3.3 Frequency analysis of RSN

**Fig. 8:**
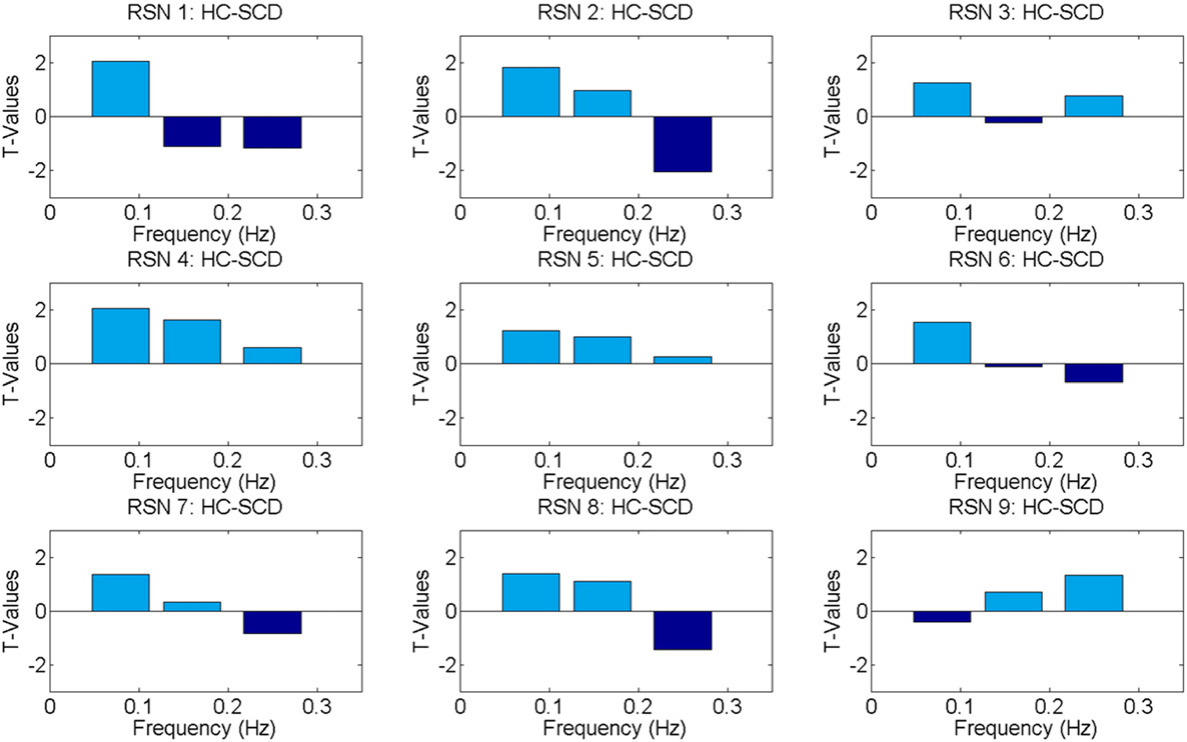
Bar plots showing spectral power analysis result for each RSN. Frequency comparison was done by subtracting patients from controls. The T-value of this result is displayed in the bar plots. A negative T-value represents greater power observed in patients. The frequency was compared across three equally spaced bins with the ranges of 0–0.08 Hz, 0.08–0.16 Hz, and 0.16–0.25 Hz.

A frequency analysis of the RSN using GIFT is shown in Fig. 8. The power spectra between the two groups were assessed at 3 equally spaced frequency bins (0–0.08 Hz; 0.08–0.16 Hz; 0.16–0.25 Hz). Bar plots have a T-value contrast showing patients subtracted from controls. It was observed that patients' RSN1, RSN2, RSN6, RSN7, and RSN8 tend to modulate at a higher frequency compared to controls. For these networks, the frequency bin ranging from 0.16–0.25 Hz had the most power for patients. Controls had more power in the lowest frequency bin ranging from 0 to 0.08 Hz for all RSN except RSN9. Only intermittent points were found to be significant (p < 0.01) with the RSN2 having the only significant point showing patients having higher fluctuations.

### 3.4 Connectivity in cerebellum

**Fig. 9:**
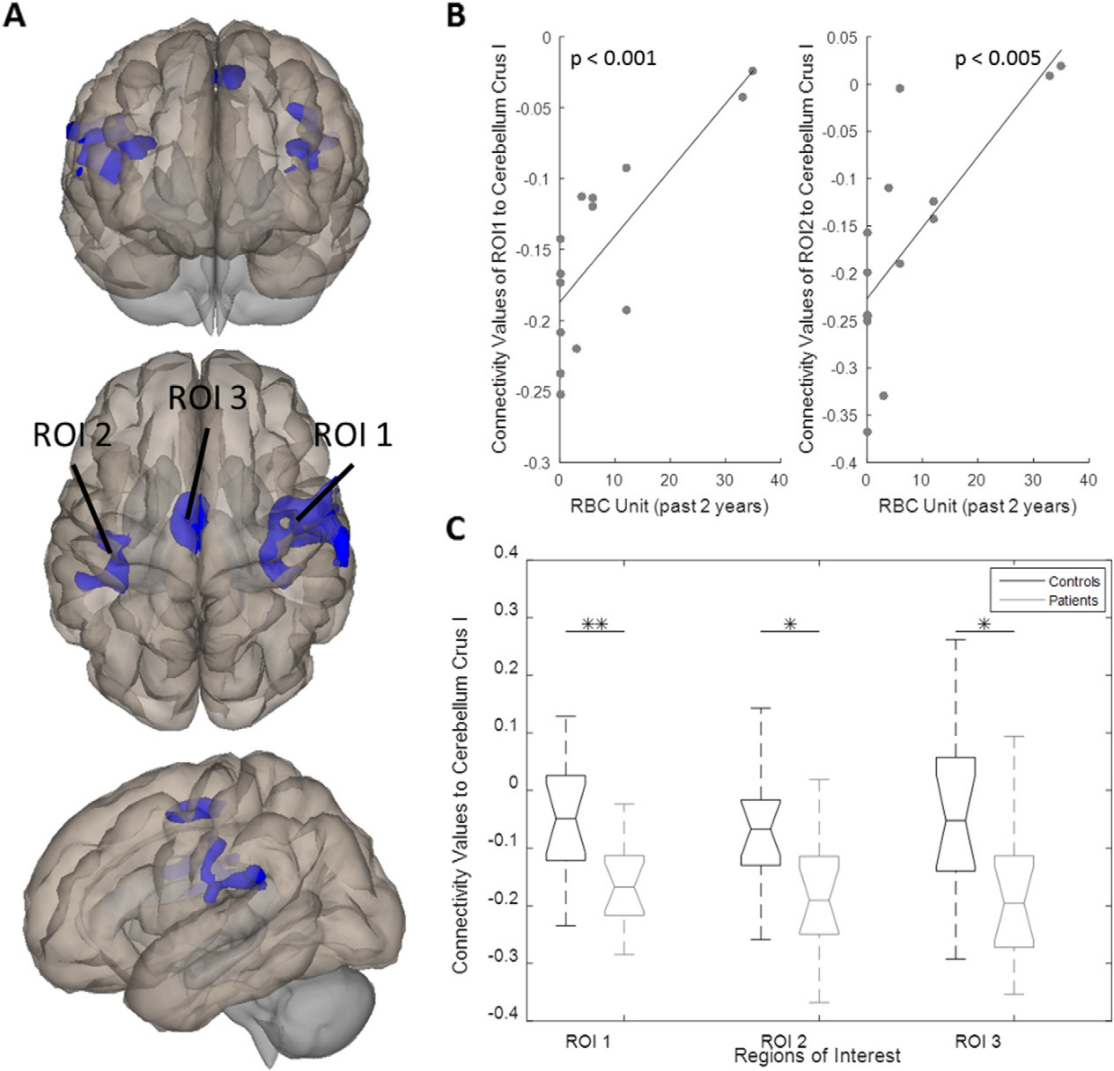
Cerebellum connectivity analysis. Seed location placed in cerebellum crus I. A. Significant clusters that have negative connectivity to seed region in patients. Three different views are shown. Regions of interest (ROI) are labeled 1–3. ROI1 included parts of the central opercular cortex, postcentral gyrus, precentral gyrus, parietal operculum, supramarginal gyrus, insular cortex, Heschl's gyrus, planum tempolare, and planum polare. ROI2 included parts of parietal and central operculum cortex, postcentral gyrus, precentral gyrus, insular cortex, planum tempolare, Heschl's gyrus, and supramarginal gyrus. ROI3 included parts of the supplementary motor cortex and precentral gyrus. B. Scatter plots of significant correlations with clinical variable red blood cell units. The linear trend line of ROI1 had p < 0.001 and the linear trend line of ROI2 had p < 0.005. Both relationships showed reduced magnitude of negative connectivity related to increased red blood cell units. C. Box plot showing differences of negative connectivity values between patients and controls. Patients all showed significant negative connectivity in each region compared to controls. The * shows significance where p < 0.05, and the ** shows significance where p < 0.005.

CONN functional connectivity toolbox was used to perform an ROI to voxel group level analysis with the seed placed in the right cerebellum Crus I. This seed location was chosen to further explore the cerebellum's influence on resting state in SCD patients and based on significant differences observed in ROI to ROI maps. The statistically significant clusters that showed negative connectivity in the patient group are shown in Fig. 9A, where each cluster was named ROI1-ROI3. The cluster size, MNI coordinates of the primary peak location, and the regions within the ROI for all ROIs are shown in Table 4. The negative connectivity values showed a significant positive correlation with the number of units of red blood cells transfused over the past two years for both ROI1 (R^2^ = 0.6215, p-value = 0.0008) and ROI2 (R^2^ = 0.5566, p-value = 0.002); seen in Fig. 9B. The amount of red blood cell transfusions is indicative of disease severity in SCD; where a zero indicates no transfusions were needed for the patient. The connectivity values to each of these three regions were also obtained for the controls. A box plot showing the differences between connectivity values between controls and patients to all ROIs is displayed in Fig. 9C. Significant differences were observed in all ROIs, where patients had greater negative connectivity in all of the regions (ROI1 p-value = 0.003, ROI2 p-value = 0.03, ROI3 p-value = 0.007).

**Table 4:** Description of significant regions with negative connectivity to the right cerebellum crus I in patients.

Region of interest	ROI 1	ROI 2	ROI 3
Cluster size (voxels)	1714	648	435
MNI coordinates (x, y, z)	(49, − 11, 21)	(− 42, − 24, 23)	(− 4, − 7, 53)
Included regions	Central opercular cortex	Parietal operculum cortex	Supplementary motor cortex
	Postcentral gyrus	Central opercular cortex	Precentral gyrus
	Precentral gyrus	Postcentral gyrus	
	Parietal operculum	Insular cortex	
	Supramarginal gyrus	Precentral gyrus	
	Insular cortex	Planum temporale	
	Heschl's gyrus	Heschl's gyrus	
	Planum temporale	Suparmarginal gyrus	
	Planum polare		

Significant clusters in patients with functional negative connectivity to the right cerebellum crus I. The cluster size is measured in number of voxels, and the primary peak location MNI coordinates are listed. Any regions within the cluster are also listed.

### 3.5 EEG-fMRI power analysis

**Fig. 10:**
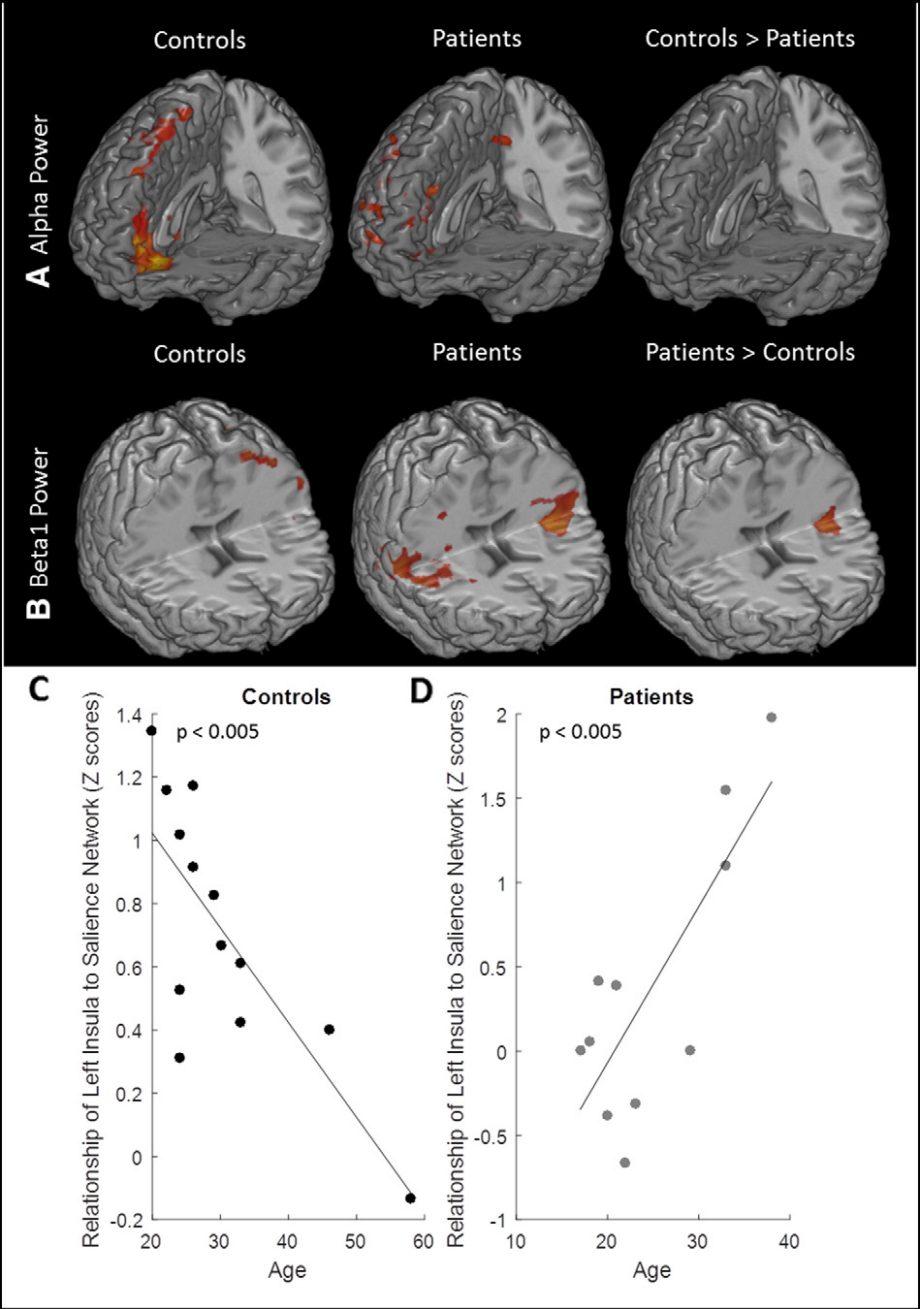
EEG-fMRI spontaneous power results. A. Power analysis group results for positive correlation to the alpha power band. Positive correlation clusters seen in prefrontal and frontal cortex in controls, and in prefrontal cortex, inferior parietal cortex, and dorsolateral prefrontal cortex in patients. Contrast images revealed there were no significant clusters in alpha band where either controls showed more connectivity or patients showed more connectivity. B. Power analysis group results for positive correlation to the beta1 power band. Positive correlation clusters seen in precentral gyrus in controls, and bilateral insula cortex in patients. Contrast images revealed that patients showed greater connectivity in the left insula. C. Scatter plot of how z-scores from left insula cluster in controls relate to age. A negative correlation was observed with p < 0.005. D. Scatter plot of how z-scores from left insula cluster in patients relate to age. A positive correlation was observed with p < 0.005. Disease severity increases with age in patients.

Fig. 10 compares spontaneous power analysis images of healthy controls and SCD patients. The medial prefrontal and frontal cortex was positively correlated with the alpha band in healthy controls. A positive correlation means that this region activates when alpha power increases. The dorsolateral prefrontal cortex (PFC), orbitofrontal PFC, and PCC activity was seen in this frequency band for SCD patients. However, no contrast image between patients and controls showed significant clusters (Fig. 10A). The beta1 power band had a positive correlation with bilateral insula cortex activation in SCD patients; this was not seen in healthy controls. A contrast image showed that the left insula had significantly greater activity in the beta1 band in patients (Fig. 10B). The left insula activation was taken as a ROI to extract z-scores from back-projected salience network maps from all subjects, please see Methods Section 2.10 for more details. There was a significant negative correlation between z-scores and age in controls (R^2^ = 0.5927, p-value = 0.002), shown in Fig. 10C, and a significant positive correlation between z-scores and age in patients (R^2^ = 0.6337, p-value = 0.003), shown in Fig. 10D.

### 3.6 EEG-fMRI microstate analysis

**Fig. 11:**
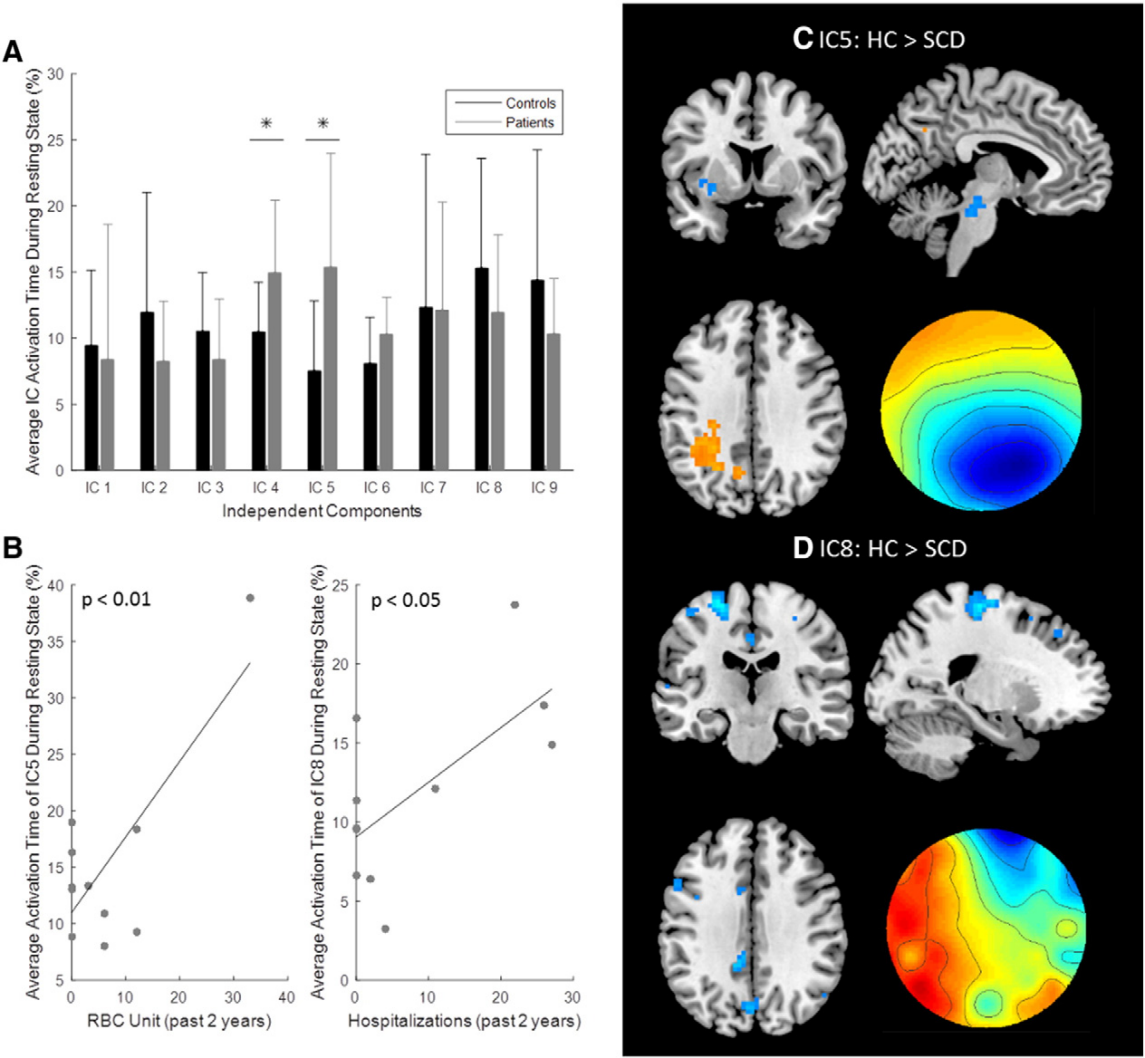
EEG-fMRI microstate analysis. A. Bar plot of the average activation time of each microstate or independent component (IC) for controls and patients. The * shows significance where p < 0.05, and the standard deviation is displayed using error bars. B. Scatter plots of significant correlations with clinical variables. The average activation time of microstate 5 (IC5) was positively correlated with red blood cell (RBC) units with p < 0.01; the linear trend line is also plotted. The average activation time of microstate 8 (IC8) was positively correlated with the number of past hospital visits with p < 0.05; the linear trend line is also plotted. C. The contrast image of microstate 5. The yellow color indicates clusters where controls had greater connectivity and the blue color indicates clusters where patients had greater connectivity. Controls had greater connectivity in the left parietal cortex, and patients had greater connectivity in midbrain, insular cortex, amygdala, superior temporal gyrus, hippocampus and putamen. The topography of the microstate is shown in the lower right hand corner. D. The contrast image of microstate 8. Patients had greater connectivity in the supplementary motor area, precentral gyrus, postcentral gyrus, dorsolateral prefrontal cortex, precuneus, and midcingulate cortex. The topography of the microstate is shown in the lower right hand corner.

Group microstate results are shown in Fig. 11. A total of nine microstates were found for this group of subjects. Two microstates had significantly greater average activation time in patients compared to controls, these included microstate 4 (p-value = 0.04) and microstate 5 (p-value = 0.02), shown in Fig. 11A. The average activation time was compared to the clinical variables, and two significant relationships were found (Fig. 11B). Microstate 5 had a positive correlation between activation time and the unit of red blood cells transfused in the past 2 years (R^2^ = 0.5952, p-value = 0.03). Microstate 8 had a positive correlation between activation time and the number of hospitalizations in the past 2 years (R^2^ = 0.402, p-value = 0.04). These two microstates were further examined by analyzing contrast images displayed in Fig. 11C–D. The controls had significant clusters in the left inferior parietal cortex and precuneus for microstate 5, where the patients had significant clusters in left insula, left putamen, amygdala, left superior temporal gyrus, hippocampus, and the brainstem (Fig. 11C). Microstate 8 had no significant clusters for greater connectivity in controls; however, patients showed significant clusters in the supplementary motor area, left precentral gyrus, left primary somatosensory cortex, dorsolateral PFC, precuneus, and midcingulate cortex (Fig. 11D).

## 4 Discussion

The multimodal non-invasive imaging utilizing simultaneous EEG and fMRI was feasible in patients with SCD, without causing any discomfort or adverse events due to the procedure. To our knowledge, this is the first multimodal fMRI-EEG study on resting state alterations in patients with SCD. We assessed RSN connectivity using fMRI analysis techniques and EEG-informed fMRI analysis methods. In particular, EEG dynamics enhanced our results by identifying additional pain processing areas active in patients, and detecting enriched temporal information regarding RSN fluctuations due to the high temporal resolution of EEG.

### 4.1 Imagining modalities

Chronic pain has been studied using fMRI and EEG as functional imaging modalities. fMRI studies have indicated that chronic pain patients have altered frequency activity in PFC, thalamus, and DMN (Apkarian et al., 2005, Lee and Tracey, 2013). In SCD patients, fMRI has demonstrated decreased BOLD signal magnitude and greater connectivity to nociceptive regions for patients with high pain (Darbari et al., 2015, Zou et al., 2011). The high spatial resolution of fMRI allows accurate localization of functional areas; however, due to limited temporal resolution, fMRI results are limited because they cannot describe the intricate temporal patterns of activation areas (He et al., 2011). EEG studies in chronic pain patients and SCD patients have shown altered responses to evoked stimuli (Colombatti et al., 2015, Flor et al., 2004). EEG can better describe temporal dynamics of the pain response, but has limited spatial resolution, so determining functional areas related to the response can be challenging (He et al., 2011). However, by combining EEG and fMRI we can better recover “where” and “when” a functional area was active. EEG-fMRI was studied in acute pain, where EEG regressors helped explain additional variance (Christmann et al., 2007, Mobascher et al., 2009). In our study, we used EEG-fMRI to analyze chronic pain. Our EEG-fMRI analysis revealed additional frequency information about DMN and saliency network, as well as show additional activation in pain processing areas in patients. These results indicate that combining these two modalities reveals more information about neural signals than using either method alone.

### 4.2 Pain areas

The most consistent regions associated with nociception across studies include the primary somatosensory cortex, secondary somatosensory cortex, anterior cingulate cortex, insular cortex, PFC, thalamus, basal ganglia, PAG, and cerebellum (Apkarian et al., 2011, Apkarian et al., 2005, Lee and Tracey, 2013, Shao et al., 2012, Tracey et al., 2002). Different types of chronic pain patients have shown abnormal activity in the striatum, insular cortex, basal ganglia, and PFC when compared to controls (Casey et al., 2003, Jääskeläinen et al., 2001, Napadow et al., 2010, Wood et al., 2007). SCD patients associated with high pain intensity were found to have greater connectivity to pronociceptive areas such as anterior cingulate cortex, primary somatosensory cortex, secondary somatosensory cortex, midcingulate cortex, and insula (Darbari et al., 2015). The dorsolateral PFC has also been implicated in pain processing and has been shown to reflect pain characteristics in chronic back pain patients (Apkarian et al., 2004, Graff-Guerrero et al., 2005, Lorenz et al., 2003). Our fMRI analysis showed that SCD patients have increased connectivity in the insular cortex, basal ganglia, and PAG regions to various RSN. Furthermore, our EEG-fMRI analysis revealed additional areas of connectivity for pain processing including the primary somatosensory cortex and dorsolateral PFC. The increased connectivity in these regions could be a result of the chronic pain caused by SCD and supports the theory that central mechanisms are involved in sickle pain (Darbari et al., 2015).

### 4.3 Resting state network connectivity

We were able to identify nine RSN in patients and controls using ICA, many of which were similar to a previous study using resting state fMRI with SCD patients (Darbari et al., 2015). Differences within these RSN were found using both traditional fMRI methods as well as using EEG-fMRI analysis. Several RSN with altered connectivity were also shown to have some relationship with clinical measures of chronic pain history or disease severity.

#### 4.3.1 Default mode network

The DMN function relates to internal processes including mind-wandering, social cognition, emotional processing, and other stimulus-independent thoughts (Heine et al., 2012, Lee et al., 2013, Van den Heuvel and Hulshoff Pol, 2010). The anterior DMN had significantly reduced active voxels in patients compared to controls. Additionally, both DMN components showed increased connectivity to sensory related regions in patients. This has been observed in other chronic pain patients (Baliki et al., 2014, Loggia et al., 2013). EEG-fMRI analysis also showed the DMN had increased connectivity to sensory regions. While the results for alpha band showed reduced DMN activity, activity was also shown in dorsolateral PFC. Microstate 5 had increased connectivity in inferior parietal cortex, a DMN node, where patients had increased connectivity to several regions associated with pain processing. Additionally, the activation time of microstate 5 positively correlated with the amount of red blood cell transfusions. Microstate 8 showed the precuneus was connected to the primary somatosensory cortex, dorsolateral PFC, and other sensory regions in patients. Activation time of microstate 8 had a positive correlation to the number of hospitalizations. These results could indicate that the DMN in patients do have increased connectivity to sensory/pain related areas and that the activation time of the altered DMN microstates reflect the disease severity of the patient. These findings in reduced DMN activity in both our fMRI analyses and EEG-fMRI analyses are in agreement with other studies of chronic back pain using fMRI (Baliki et al., 2008). Additionally, we found the DMN had increased connectivity to sensory related areas such as supramarginal gyrus, paracentral lobule, dorsolateral PFC, and primary somatosensory cortex. We believe the increased connectivity is caused by patient's chronic pain, as increased connectivity between DMN regions and sensory areas have been seen in SCD patients with high pain (Darbari et al., 2015). Chronic pain in SCD patients is suitable to be studied from the angle of DMN as the spontaneous painful sensation may produce salient percepts in the absence of exogenous input (Baliki et al., 2008, Foss et al., 2006).

#### 4.3.2 Executive control network

The right and left ECN involve external processing such as perception, nociception, and decision-making (Heine et al., 2012, Lee et al., 2013). The right and left ECN showed significantly reduced active voxels in our study. Chronic pain patients as well as SCD patients have been shown to have poor cognitive performance (Colombatti et al., 2015, Karp et al., 2006, Moriarty et al., 2011, Nes et al., 2009, Novelli et al., 2015, Zou et al., 2011). Delayed evoked potentials and altered cortical sources in SCD subjects have been observed, indicating a disease-specific alteration that may modify neural networks (Colombatti et al., 2015). One rationale theorized for reduced ECN activity in chronic pain patients is that increased pain processing is taking away resources from the ECN, which results in poor performance in executive function tasks (Glass et al., 2011). Our results support this theory because we found increased connectivity to pain processing areas, such as basal ganglia, in the ECN. Moreover, we saw a correlation to the number of active voxels in the ECN to the amount of transfused red blood cells in SCD patients.

#### 4.3.3 Salience network

The salience network is involved processing noticeable stimuli, including pain related processing, as well as being known as the switching network, where the salience network chooses to enact either the DMN or ECN based on external input (Di and Biswal, 2014, Heine et al., 2012). The salience network was shown to have stronger connectivity within patients, where both our fMRI ICA analysis and EEG-fMRI power analysis in beta1 band showed increased connectivity in the insula cortex. Functional neuroimaging studies have documented insular cortex activation is associated with different functional roles including modulation of affective-emotional processing, cognitive and affective processes during learning, and aversive pain processing (Paulus and Stein, 2006). Emotional processing could have activated insular cortex in SCD patients because mild to severe depression is frequently (44%) seen in SCD patients (Hasan et al., 2003), and insula cortex is an important structure involved in depression (Sprengelmeyer et al., 2011). Chronic pain is another candidate for insular cortex activation. Patients with chronic pain have been shown to have increased connectivity to insula cortex either between other RSN such as DMN, or between other pain processing regions (Baliki et al., 2006, Cifre et al., 2012, Darbari et al., 2015, Loggia et al., 2013, Napadow et al., 2010). We observed a relationship between age and the z-score of the left insula to the salience network in both controls and patients. The controls had a negative correlation, indicating the left insula's role in the salience network decreases with age. This most likely reflects how normal aging impairs the functional connectivity within the salience network (He et al., 2014). However, the opposite trend was seen in patients, where the left insula's role in the salience network increased with age. It is known as SCD patients age, their chronic pain increases and their disease severity worsens (Darbari et al., 2012, Platt et al., 1994, Platt et al., 1991). This means the increased involvement of left insula in salience network could be a result of increased pain severity. This is why we believe the increased connectivity observed in the salience network is reflective of chronic pain in SCD patients.

### 4.4 Altered frequency behavior in SCD

Frequency analysis of BOLD signals have shown that RSN are characterized by low frequency oscillations (< 0.1 Hz) in healthy patients (Broyd et al., 2009, Farmer et al., 2012, Garrity et al., 2007, Hlinka et al., 2010). The DMN has been shown to shift to higher frequencies in the BOLD signal for patients with neural dysfunctions, such as schizophrenia and chronic back pain (Farmer et al., 2012, Garrity et al., 2007, Otti et al., 2013). The posterior DMN in our SCD patient population showed a similar shift to higher frequencies. The cause of the frequency shift is unknown, but it could result from the altered RSN connectivity (Garrity et al., 2007). In addition to our findings, altered RSN connectivity has been observed in SCD patients for the DMN and salience network previously (Darbari et al., 2015).

Resting state EEG data have revealed that DMN is linked to traditional EEG frequency bands including alpha, delta, beta, and theta (Hlinka et al., 2010, Knyazev et al., 2012, Laufs et al., 2003a, Laufs et al., 2003b, p. 200; Laufs et al., 2006, Scheeringa et al., 2008). In our study, the DMN of healthy controls and SCD patients positively correlated with the alpha power band; however, the DMN of patients had reduced activity. The salient network positively correlated with beta1 power for SCD patients, but not for controls. These observed differences in EEG frequency bands could reflect a similar trend in low frequency oscillations, where patients have altered frequency behavior compared to controls.

### 4.5 Cerebellum connectivity

The cerebellum is known to be active during pain processing, but its actual function is not well understood (Apkarian et al., 2005, Chen, 2001, Lee and Tracey, 2013). Recent studies have tried to explore how or if the cerebellum contributes to nociception. It has been theorized certain regions within the cerebellum are related to sensory processing, while others are related to other aspects of pain such as emotional processing (Diano et al., 2016, Dimitrova et al., 2003). Our study found altered activity within the cerebellum. Patients showed increased connectivity to PAG in the cerebellum RSN. Additionally, there was significant negative connectivity differences observed in our seed based analysis, where the seed location was the right cerebellum crus I. SCD patients had greater negative connectivity compared to controls and negative connectivity had a positive correlation to the amount of transfused red blood cells. Differences in negative connectivity in cerebellum crus I have been observed in other disorders such as schizophrenia and major depressive disorder (Liu et al., 2012b, Shinn et al., 2015). These differences are thought to reflect impairment in emotional processing. The relationship observed in SCD patients could reflect how negative connectivity to each ROI, all related to sensory or emotional processing, is acting in an antinociceptive manner and reducing chronic pain and disease severity by impairing emotional and sensory processing. Disease severity increases as the magnitude of negative connectivity reduces. Irritable bowel syndrome patients have shown crus II is related to anxiety scores from these chronic pain patients (Rosenberger et al., 2012), which shows cerebellum regions can reflect emotional processing disruptions in chronic pain patients. Recently, different regions of the cerebellum have been shown to be linked to several RSN. In particular, crus I have been shown to be linked to the salience network and ECN (Borsook et al., 2008, Caulfield et al., 2015). Our study showed that both the salience network and ECN have altered connectivity in SCD patients, and so this might also explain the negative connectivity in the patients. Negative connectivity is theorized to correlate to the shortest path length, where accumulated phase delays along a pathway cause the negative functional connectivity (Chen et al., 2011). The negative connectivity observed could also be caused by the path length between the ROIs and the cerebellum. Overall, the cerebellum seems to have altered connectivity in patients that reflects disease severity.

### 4.6 Study limitations

One confounding factor in our findings of changes in network connectivity that may influence its specificity to pain is the role of medication. It is possible that medications for pain management are causing the altered connectivity and frequency behavior of RSN. However, we did not restrict patient from using their medications in order to maximize our patient population. Another limitation of our study is the small number of subjects, which lowers our statistical power and limits what conclusions we can draw due to the risk of false positives. However, recruitment is challenging in this patient population due to the irregularity of their symptoms as well as compliance. A national study of pain treatment in SCD involving 31 sties was closed early due to recruitment challenges, where only 38 patients were recruited after 6 months (Dampier et al., 2013). Another concern for our study is using healthy subjects as controls. Due to how fMRI is measured, SCD patients may have weaker BOLD signal compared to a non-SCD subject (Zou et al., 2011). However, we did not observe significant differences in the HRF between our two groups and most RSN had comparable activity; the RSN with significant differences were most likely caused by neural alterations due to SCD. Another study was able to identify similar RSN in SCD patients using ICA, showing that fMRI data can detect RSN activity in SCD patients (Darbari et al., 2015). Considering all of these limitations, our results should be interpreted with caution. However, we propose that despite these limitations EEG-fMRI offers a unique and improved approach to further explore functional brain connectivity of SCD patients. The increased temporal and spatial resolution of this method makes it desirable to further probe for markers of disease severity and potentially chronic pain that can be used to improve treatment strategies. Non-invasive imaging studies have shown it is possible to use biomarkers to objectively rate acute pain in healthy controls (Zhang et al., 2016, Wager et al., 2013). Similar techniques could be applied to chronic pain patients so that pain can be objectively measured to improve treatment and reduce the risk of opioid therapy.

### 4.7 Conclusion

Resting state alterations can lead to biomarkers that can be used to help better understand the neural pathophysiology of sickle pain. The current study identified nine RSN in SCD patients and showed that several of them have altered connectivity and temporal characteristics in the patient population. In particular, the DMN and ECN were shown to have reduced activity, while the salience network had greater connectivity in patients. Utilizing EEG-fMRI techniques enhanced our analysis because the EEG informed fMRI methods were able to describe additional temporal characteristics of the RSN as well as correlate to several clinical variables that are related to disease severity and chronic pain history. Connectivity, spatial and temporal information can all be gathered from EEG-fMRI and may be used to identify biomarkers that reflect SCD intensity to help improve pain treatment techniques.
